# NSS-ID (Nusantara speech sample Indonesian dataset): A transcribed Indonesian speech dataset covering 15 regional backgrounds, 20 speakers, and 11 sentence categories

**DOI:** 10.1016/j.dib.2026.113261

**Published:** 2026-09-13

**Authors:** Ratna Atika, Suci Dwijayanti, Bhakti Yudho Suprapto

**Affiliations:** a Department of Electrical Engineering, Sriwijaya State Polytechnic, Srijaya Negara Street, Bukit Besar, Ilir Barat I District, Palembang, 30139, South Sumatra, Indonesia; b Doctoral Program in Engineering Science, Universitas Sriwijaya, Srijaya Negara Street, Bukit Besar, Ilir Barat I District, Palembang, 30139, South Sumatra, Indonesia; c Department of Electrical Engineering, Faculty of Engineering, Universitas Sriwijaya, Jl. Palembang–Prabumulih KM. 32, Indralaya, Ogan Ilir, 30662, Sumatra Selatan, Indonesia

**Keywords:** Indonesian speech corpus, Automatic speech recognition, Regional background, Linguistic complexity

## Abstract

This data article describes an Indonesian speech corpus developed for automatic speech recognition and related speech processing research. The corpus contains 104,500 WAV recordings, consisting of 104,368 human speech recordings and 132 synthetic gap-filled recordings, with a total duration of 134.176 h. The audio files are provided in the 16 kHz, 16-bit PCM mono format. Human speech was collected in a dedicated indoor recording environment from 20 adult speakers aged 22–38 years comprising twelve male and eight female speakers representing different regional backgrounds using a BOYA BY-MM1+ microphone and Audacity 3.7.4 software. During data collection, each speaker read 209 sentences in Indonesian, with each sentence repeated 25 times using variations in intonation, volume, and speech rate. The sentences were grouped into 11 categories representing different communicative functions and levels of linguistic complexity. Each audio file is associated with a normalized and validated transcription, as well as metadata containing the speaker’s pseudonymized ID, gender, sentence category, dataset partition, recording duration, and audio properties. This corpus is partitioned using a speaker-disjoint splitting protocol with a random seed of 42 into 73,150 training files, 15,675 validation files, and 15,675 test files. 132 recordings generated using Microsoft Edge-TTS Neural are explicitly identified as synthetic gap-filling data and account for approximately 0.126% of the corpus. This repository includes audio recordings, normalized transcriptions, metadata tables, partition manifests, descriptive statistics, and validation scripts. Potential applications include the development of Indonesian ASR, speaker-independent evaluation, acoustic modeling, research on transcription and text normalization, and comparative evaluation of end-to-end and hybrid ASR architectures.

Specifications Table

**Table:** 

Subject	Computer Sciences
Specific subject area	Automatic speech recognition, speech and language processing, and low-resource Indonesian speech resources
Type of data	*Curated PCM WAV audio packaged in TAR archives; UTF-8 TXT transcripts; CSV and JSON metadata, manifests, and descriptive statistics; PNG, PDF, and SVG figures; and Python validation and reproducibility scripts*
Data collection	Speech recordings were collected from 20 Indonesian speakers reading sentences from 11 communicative categories under a predefined recording protocol with supercardioid microphone BOYA BY-MM1+ and Audacity version 3.7.4 running on Windows 10. Audio Format PCM WAV, 16 kHz, 16-bit, mono.
Data source location	*Sriwijaya State Polytechnic, Srijaya Negara Street, Bukit Besar, Ilir Barat I District, Palembang, 30,139, South Sumatra, Indonesia. Dataset assets are staged in the Hugging Face repository, while supporting documentation, scripts, and audit reports are maintained in GitHub*
Data accessibility	Repository name: Hugging Face Datasets Indonesian ASR 11-Class Dataset. Data identification number: https://doi.org/10.57967/hf/10345 Direct URL to data: https://huggingface.co/datasets/Atika88/Indonesian-ASR-11-Class-Dataset Supporting repository: https://github.com/RatnaAtika/Indonesian-ASR-11-Class-Dataset
Related research article	R. Atika, S. Dwijayanti, and B.Y. Suprapto, “Improving speech-to-text for the Indonesian language using a modified transformer,” Eastern-European Journal of Enterprise Technologies, 1(9 (139)) (2026), 78–90. https://doi.org/10.15587/1729–4061.2026.350949

## 1 Value of the Data

• The NSS-ID dataset provides a structured corpus of Indonesian speech, which includes variations in pronunciation based on the speakers’ regional backgrounds, recorded in a controlled acoustic environment, and is useful for the development of ASR and two-way communication in service robots, particularly for Indonesian as a language with limited resources.
• This dataset includes speech recordings from male and female speakers with variations in intonation, volume, speaking rate, and pronunciation based on the speakers’ regional backgrounds from several regions in Indonesia, thereby enabling further analysis of the model’s robustness to speaker and pronunciation variations.
• This dataset provides a structured Indonesian speech resource consisting of 11 sentence categories: interrogative, imperative, confirmation, scheduling, negation, rhetorical, declarative, persuasive, conditional, clarification, and exclamatory. The diversity of these sentence categories makes this dataset relevant for the development of various speech recognition-based applications, such as Indonesian-language voice interfaces, service robots, and human–robot interaction systems that operate in real time.
• The speech data was collected using a specialized supercardioid microphone with Audacity 3.7.4 software, at a sampling rate of 16 kHz, with the microphone positioned 5–10 cm in front of the sound source (the respondent’s lips).
• The NSS-ID dataset provides a structured corpus of Indonesian spoken reading, containing 104,500 WAV files with a total duration of 134.1762 h. This release consists of 104,368 human recordings and 132 explicitly labeled synthetic gap-filling recordings.

## 2 Background

Automatic speech recognition (ASR) relies on speech corpora containing audio recordings and their corresponding text transcriptions. The NSS-ID dataset was compiled to support the development of an Indonesian-language ASR system for two-way communication between humans and machines, including speech interfaces for service robots. The collection protocol included speakers from multiple regional backgrounds and prompted variations in intonation, vocal intensity, and speaking rate. Regional background is treated as a recruitment characteristic reported by the authors based on participants’ demographic records, rather than as a linguistically verified dialect label. The 15 regional backgrounds reported by the participants were recorded during the recruitment process and this release does not include independently verified dialect annotations at the recording level https://short-url.cc/Githubregionalbackground and https://short-url.cc/Hfregionalbackground. This corpus contains speech samples drawn from 209 sentences classified into 11 functional categories: interrogative, imperative, declarative, clarification, confirmation, scheduling, negation, rhetorical, exclamatory, conditional, and persuasive. A previous review reported limitations in the documentation of Indonesian speech resources and the implementation of ASR for service robots [1]. An earlier corpus, consisting of eight sentence categories, was used [2] to train and evaluate a modified Transformer-based ASR model. This data article expands upon that research by describing a comprehensive corpus that includes three additional sentence categories and provides recording protocols, pseudonymized speaker metadata, sentence category annotations, audio specifications, quality-control information, and separate training, validation, and test partitions across speakers, generated using a random seed of 42.

## 3 Data Description

The NSS-ID repository contains the Indonesian speech corpus and its supporting metadata, including orthographic transcriptions, pseudonymized speaker information, sentence-category labels, train– validation–test split manifests, audio-quality measurements, and the source data underlying the figures presented in this article. The organization and contents of the repository are summarized in Table 1. The final corpus comprises 104,500 uncompressed mono WAV files, with a total duration of 134.1762 h and a storage size of approximately 15.7 GB. All recordings are encoded using 16-bit pulse-code modulation (PCM), a sampling rate of 16 kHz, and a single audio channel. Of the total files, 104,368 are natural human-speech recordings and 132 are synthetic gap-fill recordings, representing 99.874% and 0.126% of the corpus, respectively.

**Table 1:** Organization and contents of the NSS-ID repository.

Repository path	Format and extent	Contents
README.md	Markdown	Dataset overview, audio specifications, category definitions, repository organization, access information, and usage notes.
CITATION.cff	Citation File Format	Citation metadata for referencing the NSS-ID dataset.
data/audio_shards/audio_shards_manifest.csv	CSV; one entry per audio archive	Inventory of the category-level audio archives, including archive names, file counts, sizes, and SHA-256 checksums.
data/audio_shards/by_category/	11 TAR archives containing 104,500 PCM WAV files; total size 15,623,106,560 bytes	Category-organized audio archives: Clarification.tar, Conditional.tar, Confirmation.tar, Declarative.tar, Exclamatory.tar, Imperative.tar, Interrogative.tar, Negation.tar, Persuasive.tar, Rhetorical.tar, and Scheduling.tar. The archives contain both human recordings and explicitly labelled synthetic repair files.
data/transcripts/	11 UTF-8 TXT files; 209 distinct (category, sentence_id) pairs	Category-specific reference-transcript lists. The files retain the original sentence identifiers, including documented numbering gaps. Files are provided for Clarification, Conditional, Confirmation, Declarative, Exclamatory, Imperative, Interrogative, Negation, Persuasive, Rhetorical, and Scheduling categories.
metadata/dataset_metadata_public.csv	CSV; 104,500 recording-level rows	One metadata record per WAV file, including relative path, public speaker label, transcript, sentence category, sentence identifier, dataset partition, duration, audio-format properties, and human or synthetic provenance.
metadata/transcript_sentence_inventory_public.csv	CSV; 11 category-level rows	Inventory of sentence identifiers available in each category and identifiers intentionally absent from the released prompt lists.
metadata/speaker_labels/hf_public_metadata_schema.md	Markdown	Definition of the public metadata fields, allowed values, pseudonymous human speaker labels, synthetic speaker labels, and synthetic repair-target fields.
metadata/speaker_labels/speaker_id_public_inventory.csv	CSV	Tabular inventory of the pseudonymous public speaker identifiers used in the repository.
metadata/speaker_labels/speaker_id_public_inventory.json	JSON	Machine-readable representation of the public speaker-identifier inventory.
metadata/speaker_labels/speaker_label_gender_list.csv	CSV	Mapping between pseudonymous public speaker labels and recorded gender categories.
metadata/speaker_labels/ speaker_anonymization_preparation_report.md	Markdown	Documentation of the transformation from source speaker labels to public pseudonymous identifiers.
metadata/speaker_labels/synthetic_repair_targets_public.csv	CSV; 132 repair records	Mapping between the synthetic gap-fill recordings, their source voices, intended repair targets, and the identifiers of unavailable human recordings.
splits/speaker_split_assignment_public.csv	CSV; 20 retained human speaker labels	Assignment of each pseudonymous human speaker identifier to the training, Validation, or test partition.
splits/split_summary_public.json	JSON	Machine-readable summary of the speaker-disjoint training, Validation, and test partitions generated using random seed 42.
paper/dataset_information/README.md	Markdown	Description of the dataset-summary tables, diagnostic samples, synthetic-repair records, and publication figures contained in this directory.
paper/dataset_information/dataset_stats_public.json	JSON	Overall dataset statistics, including recording counts, duration, audio properties, category coverage, and human–synthetic composition.
paper/dataset_information/per_category_public.csv	CSV	Recording count, duration, and other descriptive values summarized for each of the 11 sentence categories.
paper/dataset_information/per_speaker_public.csv	CSV	Recording count and total duration summarized by pseudonymous public speaker identifier.
paper/dataset_information/per_split_public.csv	CSV	Recording count, duration, and source composition summarized by training, Validation, and test partition.
paper/dataset_information/audio_quality_sample_public.csv	CSV; sampled diagnostic records	Audio-quality measurements for the documented sample of recordings used in the diagnostic assessment.
paper/dataset_information/synthetic_data_stats_public.json	JSON	Summary statistics for the 132 synthetic Edge-TTS repair recordings.
paper/dataset_information/synthetic_repair_rows_public.csv	CSV; 132 recording-level rows	Recording-level metadata for all synthetic repair files, allowing users to identify, retain, or exclude them.
paper/dataset_information/word_frequency_public.csv	CSV	Word-frequency counts and cumulative lexical-coverage values derived from the public transcripts.
paper/dataset_information/figures_public/	Three figures in PNG and PDF formats	Public-safe descriptive figures: F1_files_per_speaker_split_public, F3_speaker_total_duration_public, and F7_speaker_category_heatmap_public.
docs/CATEGORY_NAMING.md	Markdown	Mapping and normalization rules for Indonesian and English category labels.
docs/TRANSCRIPT_NUMBERING_NOTES.md	Markdown	Documentation of original sentence identifiers, intentional numbering gaps, and transcript-pair relationships.
docs/USAGE.md	Markdown	Instructions for accessing the TAR archives, reading the metadata, filtering synthetic files, and using the partition information.
docs/CITATION.md	Markdown	Human-readable citation instructions.
docs/DATASET_UPDATE_NOTES.md	Markdown	Record of changes made during dataset preparation and staging.
docs/HF_DATASET_INFORMATION_FINAL_REPORT.md	Markdown	Description of the dataset-information package prepared for the Hugging Face repository.
docs/HF_DATASET_INFORMATION_SELECTION.md	Markdown	Explanation of the files selected for inclusion in the repository package.
docs/hf_dataset_information_selection.csv	CSV	Tabular inventory of the selected dataset-information files.
docs/hf_dataset_information_selection.json	JSON	Machine-readable version of the dataset-information selection inventory.
manifest.csv	CSV	Repository-level inventory of deposited files and their associated package metadata.
manifest.json	JSON	Machine-readable version of the repository-level file inventory.
upload_manifest_small_files.csv	CSV; staging-control file	Inventory used to track the upload of metadata, documentation, and other non-audio files to the staging repository.
upload_summary.json	JSON; staging-control file	Summary of the staging upload process and uploaded-file counts.
LARGE_ASSETS_TO_UPLOAD_FROM_ORIGINAL_LOCATIONS.md	Markdown; staging-control file	Internal note identifying large audio assets and their intended source locations during repository assembly.

The natural-speech subset was contributed by 20 pseudonymized adult speakers aged 22–38 years, comprising 12 male and 8 female speakers. A public speaker-level and synthetic-source inventory including pseudonymous speaker IDs, source types, gender labels recorded in the metadata, dataset partitions, numbers of natural and synthetic files, and total audio durations is available in https://l1nq.com/speakerid. https://l1nq.com/hfspeakerid.

The corpus was developed using a fixed inventory of 209 Indonesian sentences. Each of the 20 speakers was assigned to produce all 209 sentences 25 times while varying intonation, tempo, and speaking rate and retaining their natural regional accents. This recording protocol yielded a target of 5225 utterances per speaker and 104,500 utterances overall. In the final corpus, 104,368 utterances are natural human-speech recordings, whereas the remaining 132 utterances are synthetic gap-fill recordings. The sentence inventory is organized into 11 categories: interrogative, imperative, declarative, clarification, confirmation, scheduling, negation, rhetorical, exclamatory, conditional, and persuasive. Sentence-level information including the sentence identifier, sentence text, category, character count, and word count is publicly available in https://l1nq.com/hfmetadata. Recording-level information is provided in https://short-url.cc/datasetinformationpublic, including the relative audio-file path, pseudonymous speaker identifier, sentence identifier, repetition number, dataset partition, recording duration, transcript, and source type indicating whether the corresponding audio file is a natural or synthetic recording.

The complete NSS-ID release uses historical speaker-disjoint assignments generated with random seed 42, without gender stratification. Each human speaker appears in only one partition. The training partition contains 14 human speakers, comprising eight female speakers and six male speakers. The validation and test partitions each contain three male human speakers and no female human speakers. Thus, all eight female human speakers are assigned exclusively to the training partition.

These historical assignments are retained to preserve continuity with the frozen nine-model benchmark. Users of the supplied manifests (splits/train.txt, splits/val.txt, and splits/test.txt) should account for the resulting gender imbalance when conducting evaluations. Although the assignments separate human speakers across partitions, the validation and test sets provide no evidence of generalization to unseen female human speakers. External evaluations following these manifests inherit the same limitation and therefore should not be presented as gender-balanced assessments of Indonesian ASR.

The speaker-disjoint train, validation, and test assignments are provided in https://sl1nk.com/speakersplit, while aggregate split-level statistics are reported in https://l1nq.com/splitsummary. Table 2 presents the number of speakers, audio files, and hours of recordings contained in each subset. The linguistic and acoustic characteristics of the target release corpus are listed in Table 3, which includes the number and duration of human and synthetic speech recordings, audio specifications, speaker label distribution, sentence categories, vocabulary size, and the non-overlapping division of training, validation, and test sets across speakers. Release target is the complete version that serves as the basis for the dataset description in the article. This scope is used to describe the number of recordings, duration, split distribution, speakers, and synthetic recordings. The target release consists of 104,500 WAV files with a total duration of 134.1762 h, including 104,368 human recordings and 132 synthetic gap-filling files. The transcripts and recordings contain 907,972 token occurrences, which correspond to 714 normalized word types. Supporting data is available via the following link https://sl1nk.com/datasetstats, https://l1nq.com/wordfrequency, and https://sl1nk.com/sentence.

**Table 2:** Distribution of speakers, audio files, and recording duration across the dataset subsets.

Dataset subset	Female human speakers	Male human speakers	Number of audio files	Recording duration (h)
Training	8	6	73,150	94.943
Validation	0	3	15,675	20.297
Test	0	3	15,675	18.936
**Total**	**8**	**12**	**104,500**	**134.176**

Note: Speaker counts refer to human participants. All female human speakers are assigned to the training partition, while the validation and test partitions each contain three male human speakers. Synthetic voice identities are excluded from these speaker counts.

**Table 3:** Composition of the NSS-ID release target.

Characteristic	Value
Total audio files	104,500
Human recordings	104,368 (99.8737%)
Synthetic gap-fill recordings	132 (0.1263%)
Total duration	134.1762 h
Human-recording duration	134.0005 h
Synthetic-recording duration	0.1757 h (632.52 s)
Mean file duration	4.6223 s
Audio format recorded in metadata	WAV; 16-kHz; 16-bit PCM; mono
Human public speaker labels	20
Male/Female public metadata labels	12/8
Synthetic source labels	18: nine Ms and nine Fs labels
Communicative categories	11
Files per category	9500
Distinct (category, sentence_id) pairs	209
Word types	714
Train/ Validation /test files	73,150/15,675/15,675
Audio archive size	15.623 GB, approximately 14.55 GiB

A total of 132 gap-filling recordings synthesized using Microsoft Edge-TTS Neural have been documented in the repository. In a corpus consisting of 104,500 audio files, these recordings account for approximately 0.126% of the dataset and have a combined duration of 632.52 s (0.1757 h). This synthetic subset consists of 122 training files, 8 Validation files, and 2 test files. This subset includes 2 declarative recordings, 9 clarifying recordings, 16 conditional recordings, 29 confirmatory recordings, 11 negative recordings, 4 scheduling recordings, 15 imperative recordings, 16 persuasive recordings, 17 rhetorical recordings, 3 exclamatory recordings, and 10 interrogative recordings. Of these recordings, 73 were generated using the male voice id-ID-ArdiNeural and 59 using the female voice id-ID-GadisNeural; speaker cloning was not applied. Aggregate statistics by dataset partition and sentence category are available in https://l1nq.com/persplit and https://l1nq.com/category. respectively. Statistics on synthetic voices and their correction targets are provided in https://short-url.cc/syntheticdatastatspublicjson and https://short-url.cc/syntheticrepairtargetspubliccsv. Detailed metadata for individual synthetic recordings is available in https://short-url.cc/syntheticrepairrowspubliccsv.

The amount of WAV files linked to each pseudonymized speaker ID is shown in Fig. 1, along with the matching training, validation, or testing subset. WAV files are distributed across the 20 pseudonymous speakers in the test, evaluation, and fixed speaker-disjoint subsets. Training (blue), validation (orange), and test (green) are the colors that represent the designated subset. The similar file counts indicate comparable recording contributions across speaker labels. However, gender representation differs across partitions: all female human speakers are assigned to the training partition, while the human speakers in the validation and test partitions are exclusively male. Fig. 2 illustrates the average time of WAV files across the 11 sentence classifications. Bars denote the mean durations specific to each group, whilst error bars signify ±1 standard deviation. Persuasive statements exhibit the greatest average duration (6.43 s), whereas urgent statements show the least (2.96 s), indicating variance in utterance length according on category. Table 4 shows information about the total recording duration contributed by each speaker among the 20 individuals using pseudonyms. The duration per speaker ranges from 5.62 h for M1 to 7.67 h for M6, indicating moderate variation in speaking duration even though the number of audio files per speaker is nearly identical. The average sentence length across all 11 categories shown in Table 5 is presented as characters per sentence and words per sentence. Persuasive sentences have an average length of 107.1 characters and 15.4 words, while imperative sentences have an average length of 38.4 characters and 6.1 words.

**Fig. 1:**
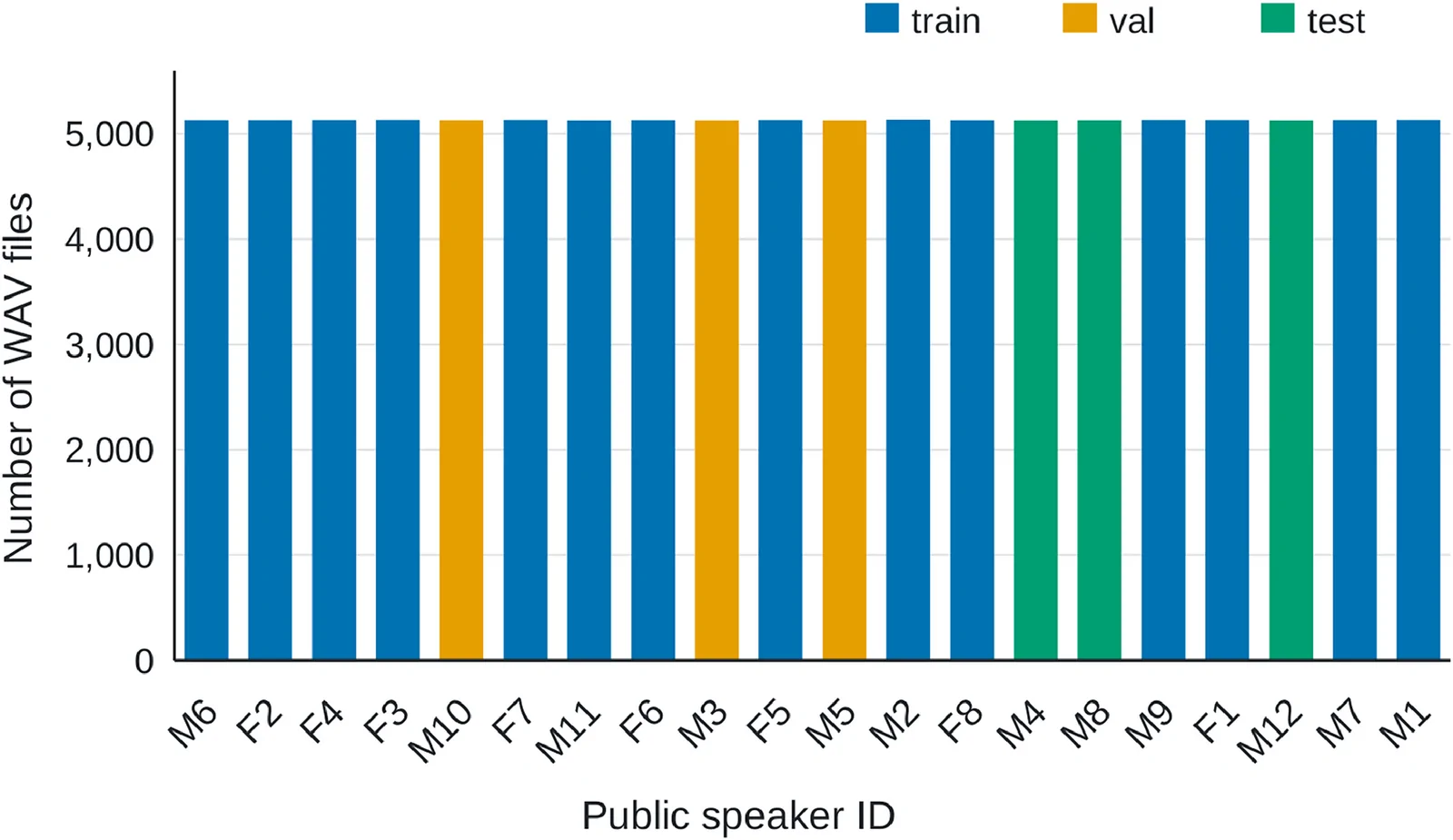
Per-speaker file count by split.

**Fig. 2:**
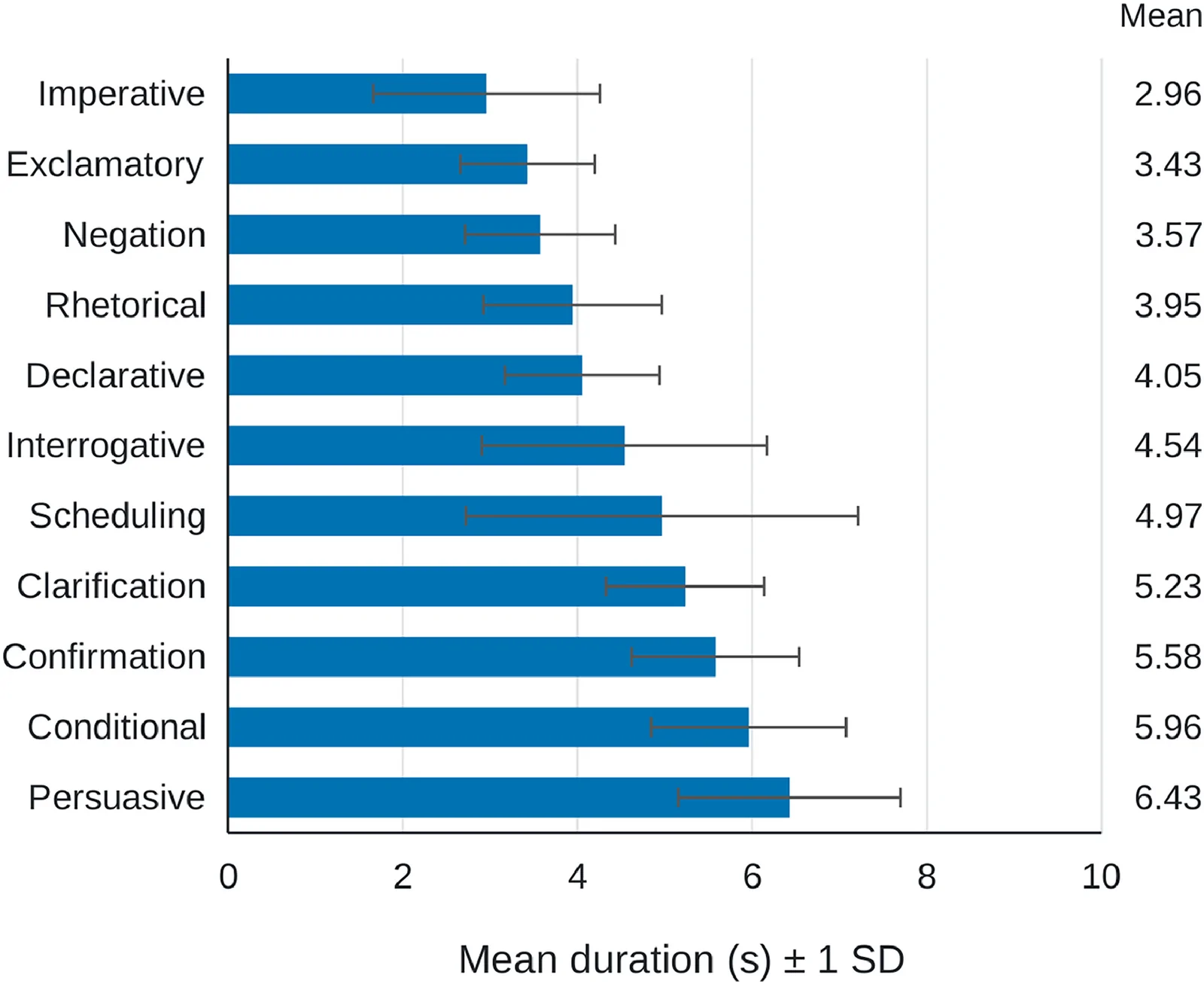
File duration per sentence category.

**Table 4:** Total recording duration for each pseudonymous speaker.

No.	Speaker ID	Gender	Split	Number of audio files	Duration (s)	Recording duration (h)
1	F1	Female	Train	5219	22,260.8175	6.1836
2	F2	Female	Train	5218	27,972.1160	7.7700
3	F3	Female	Train	5217	25,894.6877	7.1930
4	F4	Female	Train	5217	26,816.7324	7.4491
5	F5	Female	Train	5221	23,675.0672	6.5764
6	F6	Female	Train	5221	24,967.1459	6.9353
7	F7	Female	Train	5211	25,010.0250	6.9472
8	F8	Female	Train	5219	23,316.6634	6.4769
9	M1	Male	Train	5208	20,707.2655	5.7520
10	M2	Male	Train	5210	23,436.1180	6.5100
11	M3	Male	Validation	5223	24,048.2933	6.6801
12	M4	Male	Test	5225	23,182.6993	6.4396
13	M5	Male	Validation	5222	23,676.9665	6.5769
14	M6	Male	Train	5218	28,323.6758	7.8677
15	M7	Male	Train	5215	21,246.6444	5.9018
16	M8	Male	Test	5223	22,962.0589	6.3783
17	M9	Male	Train	5213	22,540.8152	6.2613
18	M10	Male	Validation	5222	25,315.8294	7.0322
19	M11	Male	Train	5221	25,036.4322	6.9546
20	M12	Male	Test	5225	22,011.9246	6.1144
**Total**	**20 speakers**	**8 Female; 12 Male**	**—**	**104,368**	**482,401.9782**	**134.0005**

**Table 5:** Mean sentence length across the 11 sentence categories.

Sentence category	Mean characters per sentence	Mean words per sentence
Imperative	38.4	6.1
Exclamatory	45.7	6.4
Interrogative	46.4	6.8
Negation	53.5	7.2
Scheduling	57.9	8.3
Declarative	60.5	8.1
Rhetorical	63.6	8.6
Clarification	65.5	9.7
Confirmation	72.0	10.2
Conditional	79.5	11.1
Persuasive	107.1	15.4

Fig. 3 shows the rank-frequency distribution on logarithmic axes of the 714 normalized word types found in the transcript corpus. The curve has a Zipf-like pattern, with a lengthy tail of increasingly less frequent word types after a small number of words that occur frequently. As shown by the distribution in Fig. 3, Table 6 presents in detail the exact frequencies, relative percentages, and cumulative percentages of the 30 most frequently occurring words. The complete 714-row source table is available in https://l1nq.com/githubwordfrequency.

**Fig. 3:**
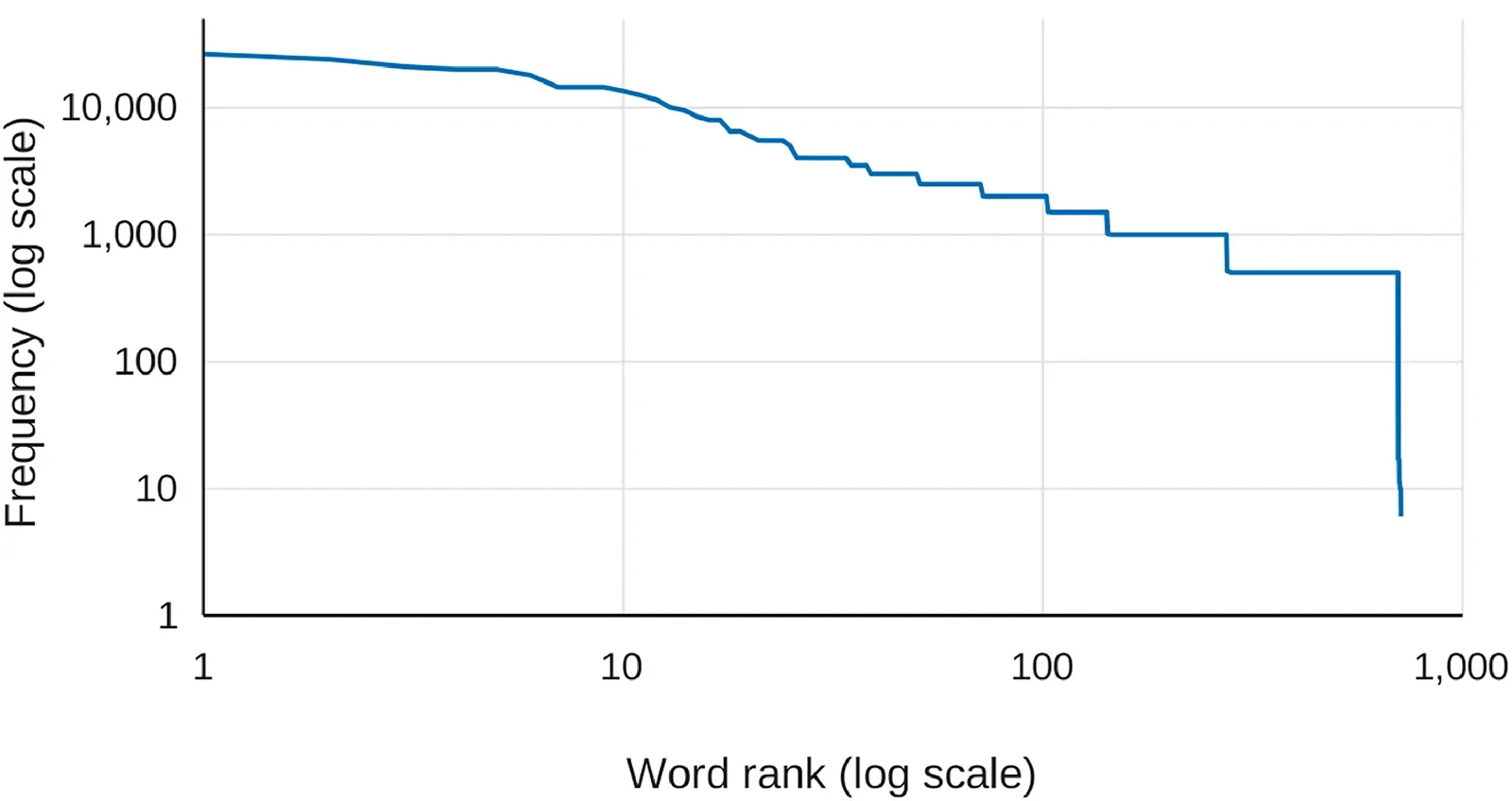
Word-frequency distribution.

**Table 6:** Thirty most frequent word types.

Rank	Word	Frequency	Share (%)	Cumulative share (%)
1	yang	26,500	2.923	2.923
2	kamu	24,000	2.648	5.571
3	apakah	21,017	2.319	7.890
4	saya	20,000	2.206	10.096
5	ini	20,000	2.206	12.302
6	jika	18,016	1.987	14.290
7	di	14,517	1.601	15.891
8	untuk	14,500	1.600	17.491
9	sudah	14,500	1.600	19.090
10	kita	13,521	1.492	20.582
11	akan	12,511	1.380	21.962
12	tolong	11,517	1.271	23.233
13	lebih	10,010	1.104	24.337
14	dengan	9523	1.051	25.388
15	ke	8500	0.938	26.325
16	bisa	8010	0.884	27.209
17	tidak	8000	0.883	28.092
18	kampus	6500	0.717	28.809
19	itu	6500	0.717	29.526
20	jadwal	6000	0.662	30.188
21	semua	5517	0.609	30.796
22	palembang	5500	0.607	31.403
23	belajar	5500	0.607	32.010
24	dan	5500	0.607	32.616
25	tugas	5000	0.552	33.168
26	waktu	4011	0.442	33.611
27	banyak	4010	0.442	34.053
28	ada	4006	0.442	34.495
29	sistem	4000	0.441	34.936
30	mahasiswa	4000	0.441	35.377

Category-level file counts and recording durations are presented in Table 7. There are 9500 files in each of the 11 sentence categories. The corresponding mean file durations range from 2.9603 to 6.5202 s, and the total recording duration varies from 7.8119 h for Imperative to 17.2061 h for Persuasive https://l1nq.com/category. Fig. 4 and Table 8 present the composition of the release target and training datasets. The validations and test partitions each contain 15,675 files from three human speakers, while the training partition contains 73,150 files from fourteen human speakers. The durations of each partition are 94.9437, 20.2969, and 18.9357 h, respectively. There are 122 files in the training partition, 8 files in the validation partition, and 2 files in the test partition. For detailed data on synthetic recordings by voice identity, see https://short-url.cc/perspeakerpublicCsV, https://l1nq.com/persplit and https://sl1nk.com/speakersplit.

**Table 7:** File count and recording duration by sentence category in the NSS-ID release target.

Sentence category	Files	Duration (s)	Duration (h)	Mean duration (s)
Clarification	9500	49,711.9233	13.8089	5.2328
Conditional	9500	57,493.4681	15.9704	6.0519
Confirmation	9500	53,262.2190	14.7951	5.6065
Declarative	9500	38,513.0637	10.6981	4.0540
Exclamatory	9500	32,552.1659	9.0423	3.4265
Imperative	9500	28,122.6942	7.8119	2.9603
Interrogative	9500	42,788.8383	11.8858	4.5041
Negation	9500	33,954.5354	9.4318	3.5742
Persuasive	9500	61,942.1090	17.2061	6.5202
Rhetorical	9500	37,489.4433	10.4137	3.9463
Scheduling	9500	47,204.0380	13.1122	4.9688
**Total**	**104,500**	**483,034.4981**	**134.1762**	**4.6223**

**Fig. 4:**
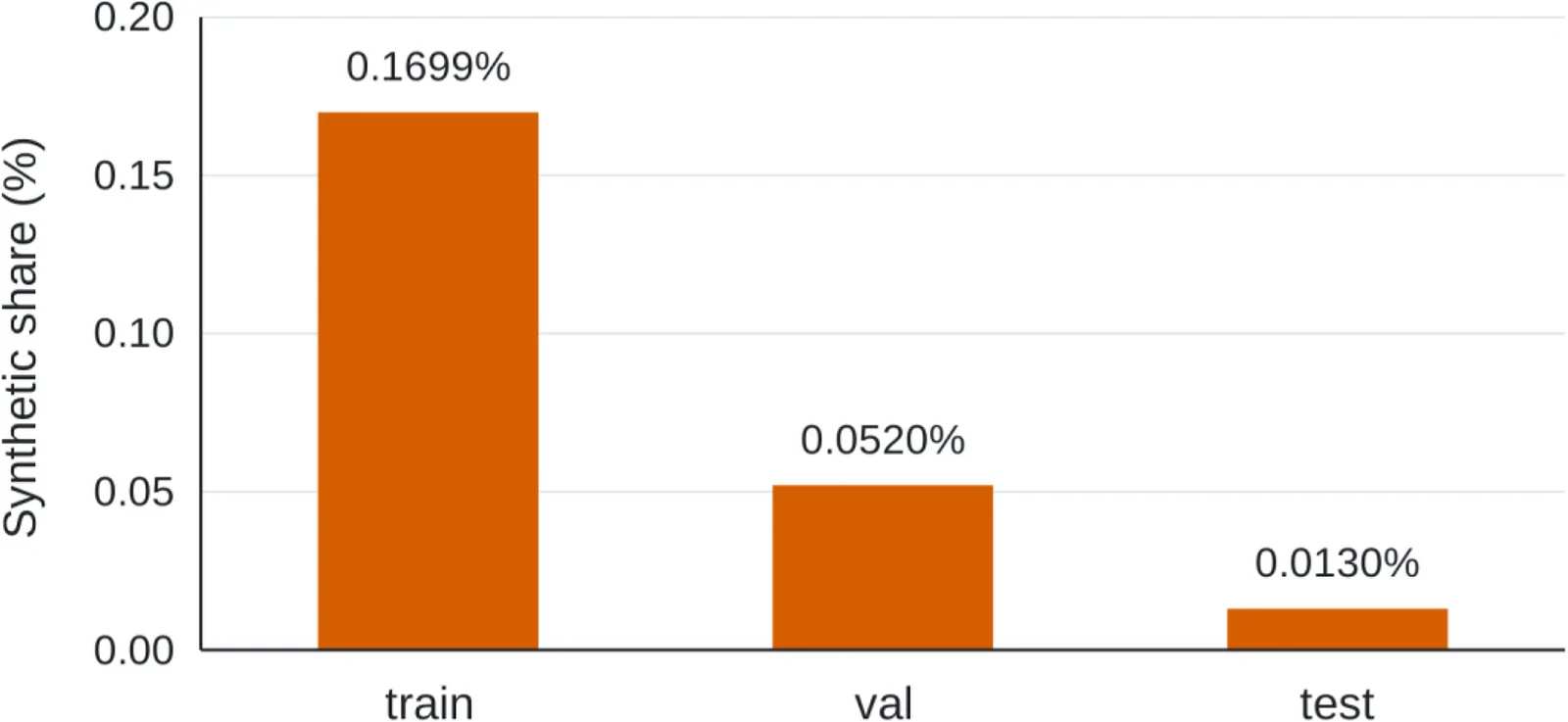
Synthetic Edge-TTS gap-fill share by split.

**Table 8:** Human and synthetic file composition by release-target partition.

Partition	Human speakers	Total files	Human recordings	Synthetic files	Duration (h)	Synthetic share (%)
Train	14	73,150	73,028	122	94.9437	0.1668
Validation	3	15,675	15,667	8	20.2969	0.0510
Test	3	15,675	15,673	2	18.9357	0.0128
**Total**	**20**	**104,500**	**104,368**	**132**	**134.1762**	**0.1263**

Table 9 shows the distribution of 132 artificial gap-filling files for each of the 11 sentence types. The Declarative category has two synthetic files, while the Confirmation category has twenty-nine. The percentage of synthetic files in each category varies from 0.0211% to 0.3053% because each category has 9500 files.

**Table 9:** Synthetic-file distribution by sentence category in the NSS-ID release target.

Sentence category	Total files	Human recordings	Synthetic files	Synthetic share of category (%)
Clarification	9500	9491	9	0.0947
Conditional	9500	9484	16	0.1684
Confirmation	9500	9471	29	0.3053
Declarative	9500	9498	2	0.0211
Exclamatory	9500	9497	3	0.0316
Imperative	9500	9485	15	0.1579
Interrogative	9500	9490	10	0.1053
Negation	9500	9489	11	0.1158
Persuasive	9500	9484	16	0.1684
Rhetorical	9500	9483	17	0.1789
Scheduling	9500	9496	4	0.0421
**Total**	**104,500**	**104,368**	**132**	**0.1263**

https://sl1nk.com/syntheticdatastats and https://sl1nk.com/syntheticrepair. An example of a Mel spectrogram for one human speech recording from each of the eleven sentence types is shown in Fig. 5. The category, anonymised public speaker ID, and the length of the recording are all shown by the labels in the panel.

**Fig. 5:**
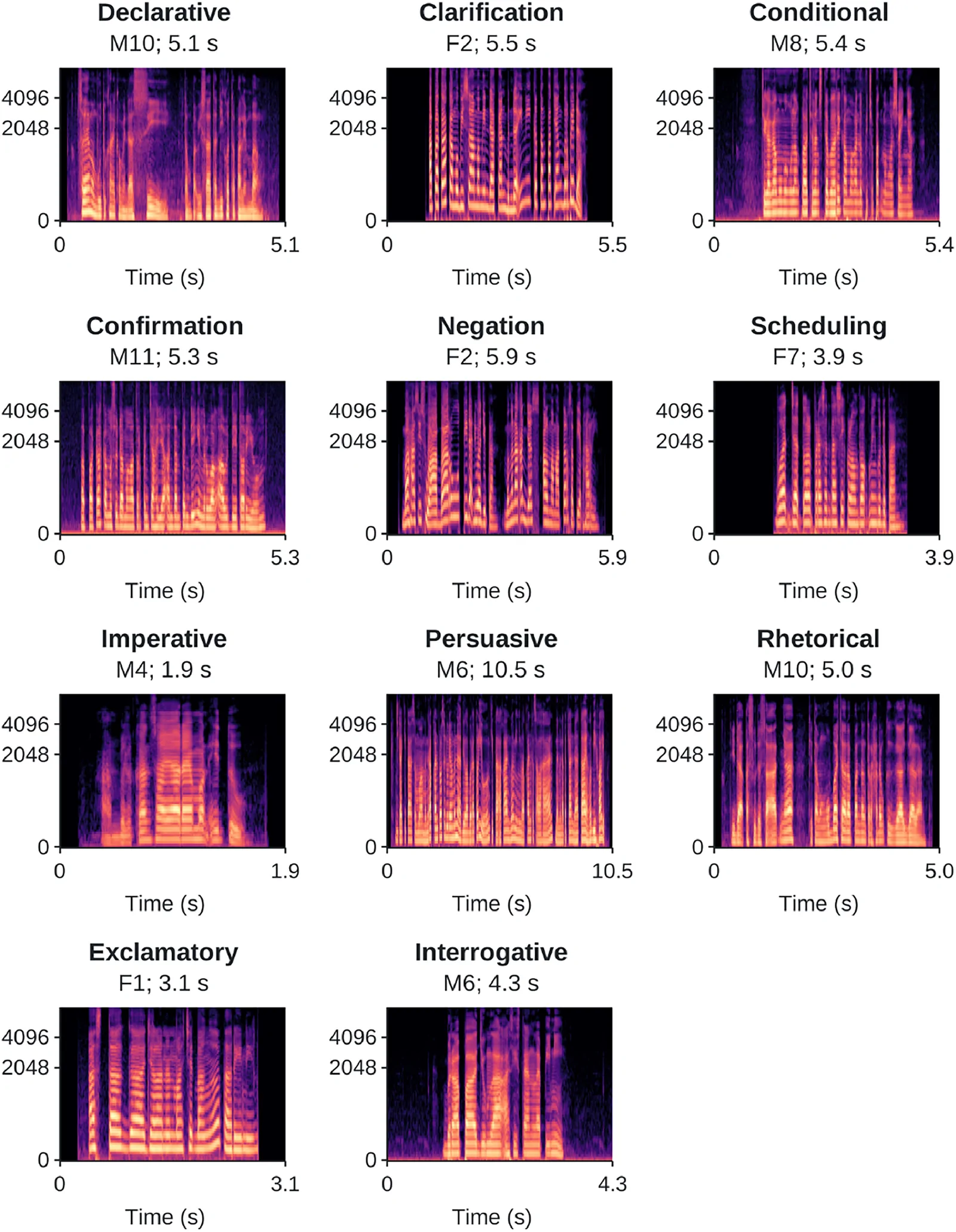
Mel-spectogram exemplars.

## 4 Experimental Design, Materials and Methods

### 4.1 Data collection process

The speech data were collected in a dedicated recording room in the Electrical Engineering Laboratory Building at Sriwijaya State Polytechnic. This room was designed to block external noise and reduce sound reflections within the room. The selected location is situated in a corner of the building, away from external noise sources. This room was constructed as an acoustic chamber lined with sound-absorbing foam. The room was constructed as an acoustic chamber lined with sound-absorbing materials. It measures 1 × 1 m, with a height of 2.5 m, and was designed with angle surfaces to minimize sound reflections. Each wall and the ceiling are lined with glass wool, followed by the acoustic foam designed to absorb sound waves and reduces echoes and internal sound reflections. The foam is a specialized “egg-crate” type selected for its ability to absorb a wide range of sound reflections, particularly mid to high-frequency sounds. The foam was installed extensively on the walls and partions of the ceiling, with no gaps to maximize sound absorption. The floor is covered with thick carpet. A dedicated sound recording room is shown in Fig. 6. The speech data were collected from male and female participants aged 22–38 who had distinctive pronunciations or accents from various regions and provinces in Indonesia. Their regions of origin included Bekasi, Bandung, Yogyakarta, Semarang, Sragen, Medan, Padang, Palembang, Bengkulu, Maluku, Baturaja, Kayuagung, Sekayu, Tanjung Enim, and Penukal Abab Lematang Ilir. These locations are collectively referred to as “regions” because they represent different administrative levels, including cities, regencies, and provinces in Indonesia.

**Fig. 6:**
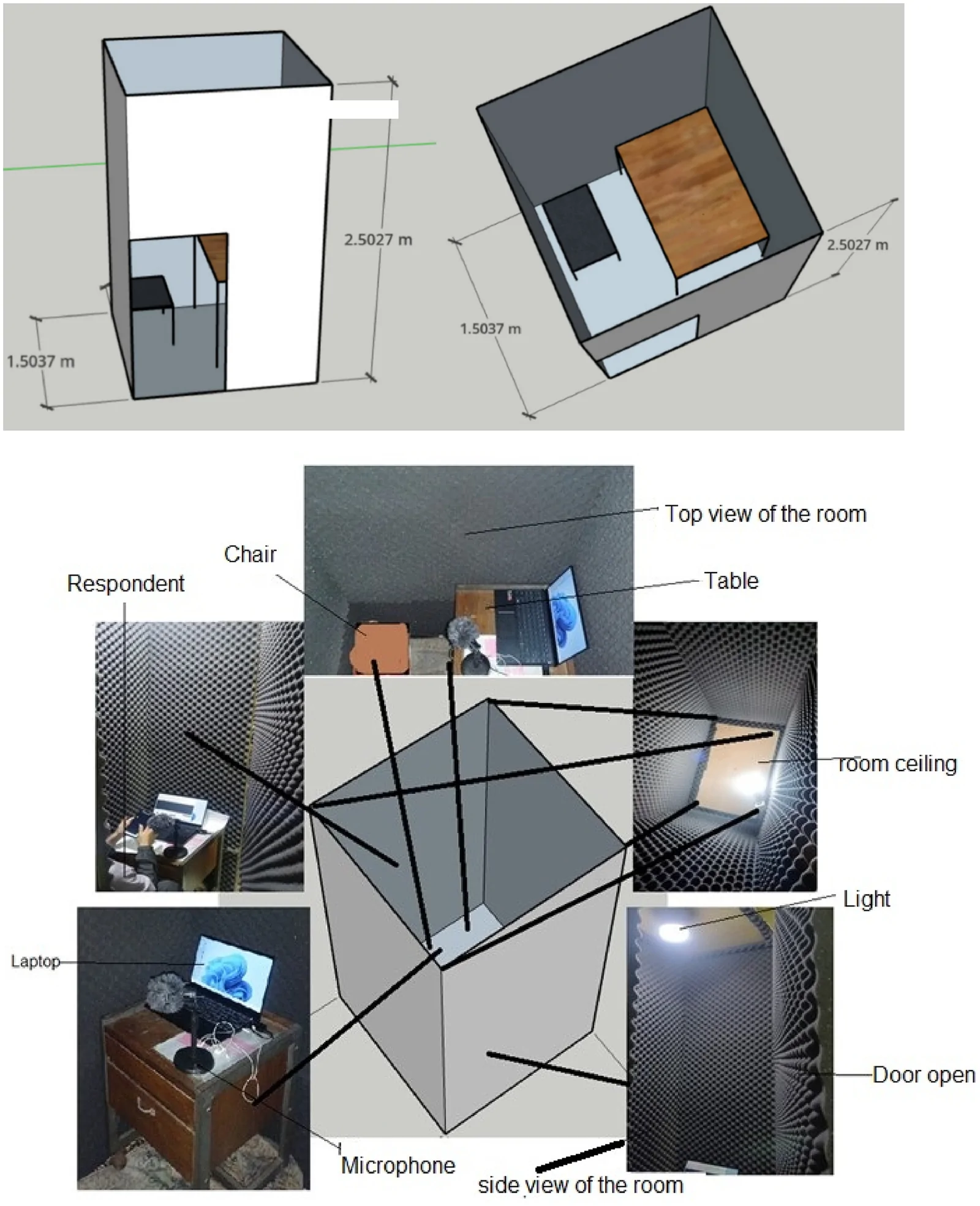
A specialized room designed for voice data recording.

### 4.2 Collection of speech-audio recordings

Speech data were recorded using a BOYA BY-MM1+ supercardioid microphone positioned approximately 5–10 cm from each participant’s mouth and Audacity version 3.7.4 running on Windows 10. The recordings were saved and exported as mono WAV files with 16-bit PCM encoding and a sampling rate of 16 kHz. During each recording session, participants read 19 sentences in sequence from the same category, and the resulting speech was initially saved as a single continuous audio file, as shown in Fig. 7. Each continuous recordings was then split at sentence boundaries using a Python-based script, resulting in 19 separate WAV files for each sentence type. The same recording and splitting procedures were applied to all 11 sentence categories.

**Fig. 7:**
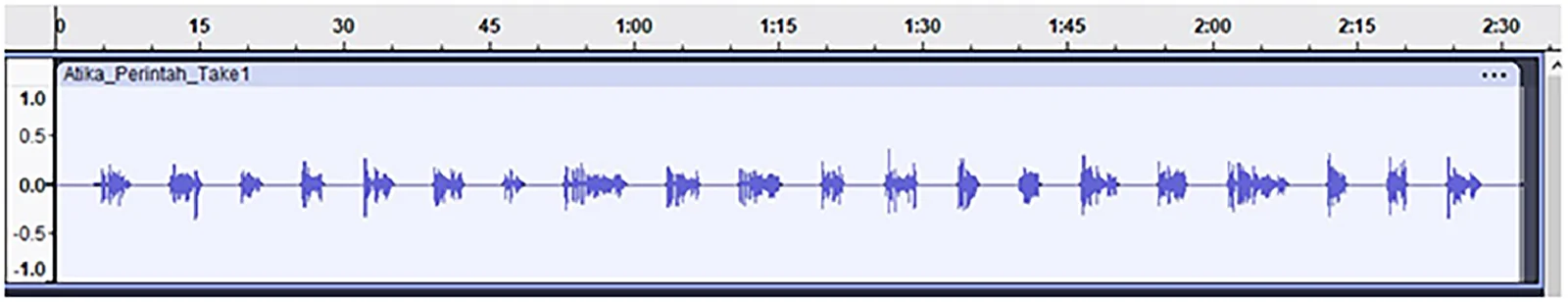
An example of raw audio recording data before it was split into 19 WAV files.

The public release contains final utterance-level WAV files rather than the original continuous pre-segmentation recordings. A retained consent/protocol sample describes category-level continuous capture followed by separation into individual files; however, the capture-time segmentation project, software/version record, silence or boundary thresholds, leading/trailing padding configuration, manual boundary-edit log, and source-to-segment manifest were not retained in the public package.

The reproducible downstream processing workflow begins with the segmented hierarchy, which consists of 11 categories, 20 preserved human-assigned labels, 25 extraction directories per category–label combination, and 20 numbered WAV files per extraction. The ‘process_paper_dataset_sota.py’ script verifies the expected inventory, detects missing, unexpected, duplicate, or non-numeric filenames, preserves the 19 IDs specified in the transcript inventory, normalizes filenames using two-digit zero-padding, and verifies the resulting hierarchy. This process copied 104,500 files and processed 5500 source runs without detecting any structurally invalid or problematic source runs during the assembly process. However, this check ensures only the consistency of the structural assembly and filenames; it does not establish the historical segmentation parameters or boundary accuracy.

The structural audit verified the category/source-label/take hierarchy, the expected numbered-file inventory, retained and omitted sentence IDs, filename normalization, missing and unexpected files, duplicate numeric IDs, and exact output counts. The audit covered 110,000 source WAV files and the 104,500-file balanced output. Sample-level acoustic diagnostics for 297 recordings, including RMS, peak amplitude, dynamic range, silence ratio, spectral centroid, and duration, are publicly available at the following resources: https://short-url.cc/audioqualitysamplepublic, https://short-url.cc/datasetstatspublicjson, and https://short-url.cc/hfdatasetinformationpublic. The 297 records constitute a descriptive diagnostic sample rather than a quality audit of all 104,500 recordings.

The 104,500-file release includes 132 explicitly flagged Microsoft Edge-TTS repair files with a combined duration of 632.52 s (0.1757 h): 122 occur in the training set, 8 in the validation set, and 2 in the test set. Row-level metadata records the synthesis engine, provider voice, synthetic public label, repair-target public label, generation-round label, and retained quality-score field. A total of 73 files use ‘id-ID-ArdiNeural’, while 59 use ‘id-ID-GadisNeural’. Of these recordings, 124 are labelled ‘v7_initial’ and 8 are labelled ‘v7_residual_fix’. The 132 stored quality scores have a minimum of 0.9101 and a mean of 0.9941. However, the original scoring implementation, model/version, normalization rule, and decision threshold were not retained. Therefore, the quality-score field is reported as historical metadata rather than as a reproducible validation metric.

Likewise, the original Edge-TTS package/service version, generation date, speech rate, pitch, volume, initial codec, conversion command, post-processing procedure, retry logic, and generated-versus-retained ledger were not recovered. Two test-set repairs use the female provider voice ‘id-ID-GadisNeural’ for the male repair-target label M8 and remain marked ‘voice_gender_matches_target=False’. A diagnostic acoustic comparison found median fundamental frequencies of 235.04 Hz and 230.56 Hz, which are close to the ‘id-ID-GadisNeural’ reference median of 233.81 Hz. These values are therefore consistent with the recorded female-provider provenance and do not support relabelling the recordings as male-voice samples. These rows are excluded from gender-stratified analyses.

### 4.3 Audio preprocessing and dataset construction

The preprocessing workflow converts category-level data sources into a standardized utterance-level audio format, the steps in this workflow are shown in Table 10 and Fig. 8.

**Table 10:** NSS-ID Preprocessing Stages.

Stage	Process	Output
**1. Sentence-inventory preparation**	Define 209 sentences across 11 categories, with 19 sentences in each category. Assign each sentence a sentence_id, canonical text, category, character count, and word count	Metadata sentences
**2. Source-audio recording**	In each recording session, a speaker consecutively reads 19 sentences from the same category, which are captured as a single source recording. This process is conducted for all 11 categories and repeated 25 times	Source recordings indexed by speaker, category, and repetition
**3. Utterance segmentation**	The long recording was split into 19 WAV files using a Python script, with each file containing a single sentence. The start and end points of each utterance were manually checked after the automatic splitting.	Utterance-level audio files
**4. File naming and identification**	Associate each segment with a pseudonymous speaker ID, sentence ID, sentence category, and repetition number.	Uniquely named and identifiable audio files
**5. Audio-format standardization**	Verify the audio format and, where necessary, convert the files to uncompressed WAV format with a 16 kHz sampling rate, 16-bit PCM encoding, and a single audio channel.	Uniformly formatted audio files
**6. Audio-boundary inspection**	Checks whether the beginning or end of a word is truncated, whether two sentences are inadvertently joined together, and whether there is excessive silence. Internal silences that are a natural part of speech are not removed.	Correctly segmented utterances
**7. Transcript validation**	Match each audio file with its canonical sentence using the corresponding sentence_id. Check for incorrect ordering, omitted words, mispronunciations, and mismatches between the audio and transcript.	Validated audio–transcript pairs
**8. Metadata generation**	Record the speaker_id, sentence_id, repetition, split, duration, and transcript.	Metadata recordings
**9. Technical quality control**	Check WAV decodability, duration, sampling rate, bit depth, number of channels, empty or corrupted files, clipping, dynamic range, silence ratio, and duplicate files.	Quality-control report and correction list
**10. Problematic-file handling**	Reprocess incorrectly segmented files from their corresponding source recordings. Re-record unusable speech where possible. Treat unavailable natural recordings as synthetic gap-fill files rather than as human recordings.	Corrected files and documented correction history
**11. Synthetic-file labelling**	A total of 132 Edge-TTS files were recorded in the synthetic file metadata, including the replaced target, the synthetic voice, and the synthesis parameters.	Synthetic manifest
**12. Dataset partitioning**	Retain the historical speaker-disjoint assignments generated using random seed 42, without gender stratification. All natural recordings from each human speaker belong to a single partition. The training partition contains eight female and six male human speakers, while the validation and test partitions each contain three male human speakers. These assignments are preserved to ensure benchmark reproducibility, with the restriction on gender coverage explicitly documented.	splits/train.txt, val.txt, dan test.txt
**13. Final integrity audit**	Verifying the consistency of metadata with the physical files, including ID uniqueness, file paths, number of recordings, total duration, category distribution, and synthetic data	Release targets ready for publication
**14. Dataset packaging**	Organize the audio files, metadata, split manifests, documentation, license, dataset version, checksums, and usage instructions for distribution through GitHub and Hugging Face.	Publicly reusable NSS-ID dataset

**Fig. 8:**
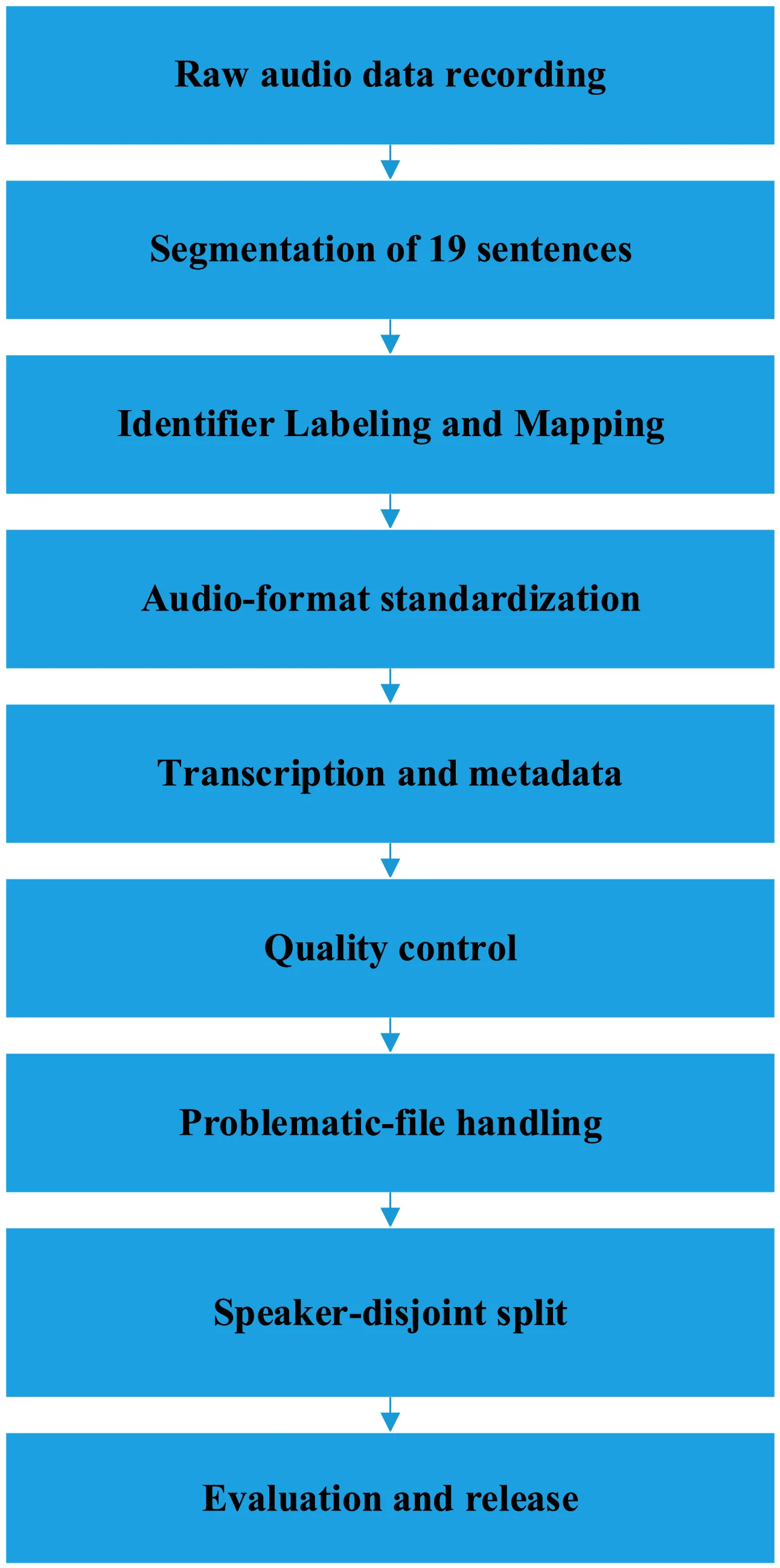
Workflow preprocessing NSS-ID dataset.

During each recording session, each speaker reads 19 sentences belonging to the same category from a predetermined set of sentences. This procedure is performed for all 11 sentence categories and repeated approximately 25 times, resulting in a notional total of approximately 209 sentences and 5225 speech slots per speaker. Each source recording is split using a Python-based processing script into 19 separate files at the utterance level, according to a predetermined sentence order. The resulting segments are then evaluated to identify incorrect boundaries, interrupted utterances, merged utterances, excessive silence, and discrepancies between the audio and the expected sentences. Each validated segment is assigned a unique filename and linked to a pseudonymous speaker identifier, a sentence identifier, a category label, and a repetition number. Audio files are evaluated for decodability and, where necessary, converted to an uncompressed WAV format with a sampling rate of 16 kHz, 16-bit pulse-code modulation (PCM), and a mono audio channel. Recording-level metadata, speaker identification, phrase identifiers, repetition numbers, dataset partitions, recording duration, and transcripts are preserved. Audio quality-control procedures include checking for missing or duplicate IDs, incorrect file paths, corrupted or empty audio, inconsistent sampling rates, unusual durations, clipping, excessive silence, segmentation errors, and inconsistencies in the transcripts. Files requiring correction are identified in the original recording and reprocessed where possible. The 132 speech slots that do not contain natural recordings are represented by clearly labeled Edge-TTS gap-filler files. These files are identified as synthetic in the recording-level metadata and described separately in the synthetic metadata file. After quality control, the recordings are allocated to the training, validation, and test partitions using a speaker-disjoint design with a random seed of 42. As a result, recordings from a given speaker appeared in only one partition. A final integrity audit verified the consistency between files and metadata, identifier uniqueness, partition membership, speaker separation, audio format consistency, corpus duration, and the match between the expected and observed number of files. The resulting release contains 104,500 WAV files with a total duration of 134.1762 h, consisting of 104,368 natural recordings and 132 explicitly labeled synthetic gap-filling files.

### 4.4 Dataset evaluation

To evaluate the technical preparedness and operational efficacy of NSS-ID, nine heterogeneous automatic speech recognition (ASR) models were tested using a static (frozen) benchmark subset of 102,544 audio recordings with an aggregate duration of 130.6548 h. The frozen benchmark is a subset of the dataset frozen at the time the nine ASR model experiments were conducted. This dataset includes only 102,544 recordings and 130.6548 h. The frozen subset and its historical speaker assignments are retained to preserve the experimental protocol underlying the reported nine-model results. Evaluation on a common dataset supports comparisons within this protocol but does not establish gender-representative performance. The difference from the released dataset is: 1956 recordings, or 3.5214 h. These recordings were not included in the frozen benchmark because, at the time the dataset was frozen, there were still rows with empty transcriptions or uncorrected transcriptions. After the metadata was corrected, these recordings were included in the target release. The benchmark composition, track-level information, reference transcriptions, and speaker-specific partitioning for training, validation, and testing (71.792; 15.376; 15.376) were established before model training. No modifications to the files or partition assignments were made during the experiment, ensuring that each model was evaluated using the same baseline dataset. The nine heterogeneous ASR models tested on this dataset consist of ViT-modified-ID, whose Transformer foundation and vision patches are described in [2]; a customized Whisper-small model [3], Conformer-CTC, which combines self-attention with convolutional processing [4], Bi-LSTM CTC, which combines bidirectional recurrent modeling with the Connectionist Temporal Classification (CTC) objective [5], the standard Transformer [6,7], the Wav2Letter-style CNN–CTC model [5,8,9], the classical HMM–GMM [5,8], the DNN–HMM hybrid system [5,9], and a three-stage GMM–HMM–DNN system in which GMM–HMM alignment is used to support subsequent neural acoustic modeling [9]. Overall, these systems represent pre-trained encoder–decoder models, end-to-end attention-based and convolutional architectures, recurrent CTC models, as well as conventional statistical–neural ASR pipelines. Because these methods differ in terms of pretraining, acoustic features, tokenization, decoding, and computational requirements, this evaluation is intended as a heterogeneous technical validation of the dataset’s utility rather than a comparison of model architectures. Fig. 9 and Table 11 present the results of the nine ASR models on the frozen NSS-ID test partition. All human speakers in this partition are male, and the test set additionally includes two explicitly marked synthetic recordings generated using a female provider voice. These results support technical validation under the historical protocol but do not establish performance on unseen female human speakers. Because sentence content overlaps across partitions, the evaluation primarily concerns the recognition of familiar sentence content spoken by held-out male human speakers.

**Fig. 9:**
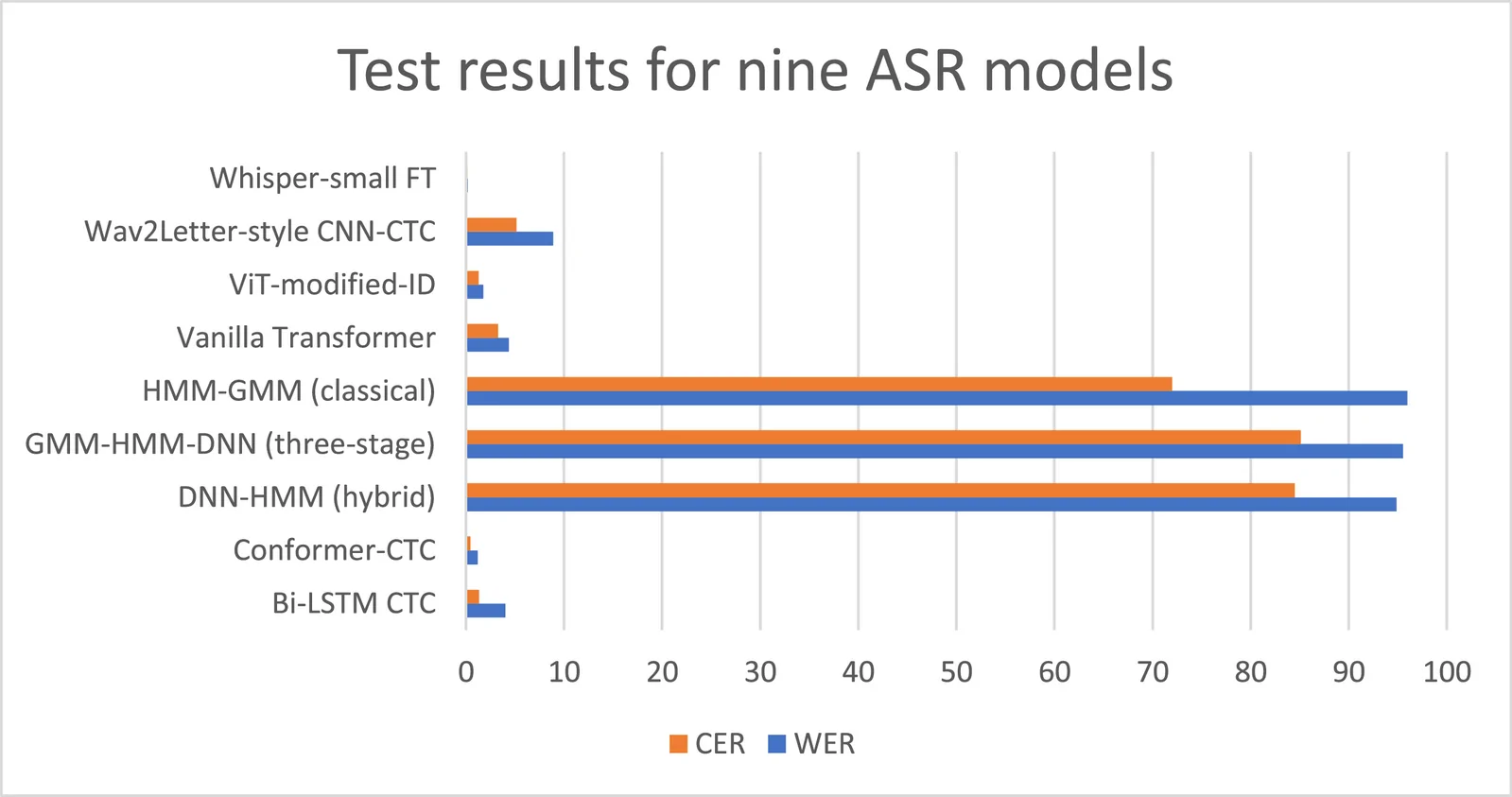
Test results for nine ASR models using the NSS-ID dataset.

**Table 11:** WER and CER results of nine ASR models on the NSS-ID dataset.

Model	WER	CER
Bi-LSTM CTC	4.012	1.322
Conformer-CTC	1.194	0.432
DNN—HMM (hybrid)	94.903	84.494
GMM-HMM-DNN (three-stage)	95.552	85.117
HMM-GMM (classical)	95.966	72.020
Vanilla Transformer	4.369	3.270
ViT-modified-ID	1.761	1.298
Wav2Letter-style CNN—CTC	8.884	5.154
Whisper-small FT	0.186	0.140

Note: All human speakers in the test set are male. The two female-voice synthetic test recordings do not provide evaluation coverage of unseen female human speakers; therefore, the reported scores should not be interpreted as gender-balanced performance estimates.

## Limitations

NSS-ID is a controlled, directed speech corpus organized around 20 pseudonymized human speaker labels; it is not a statistically representative sample of the Indonesian population. The recordings use a fixed set of prompts and a single controlled indoor recording setup; therefore, the corpus does not capture spontaneous conversations, a variety of recording devices, remote recording conditions, or the full range of real-world acoustic environments. Human public speaker labels do not overlap across the training, validation, and test sets, but prompt content overlaps across partitions. Consequently, this frozen benchmark measures recognition of known or overlapping prompts spoken by held-out human speakers, rather than generalization to completely unseen sentences, open-vocabulary speech, verified dialects, or real-world deployment conditions.

Regional background was reported during recruitment, but no independently verified, recording-level dialect labels were provided. This release contains 132 explicitly marked Edge-TTS fixes (122 in the training set, 8 in the validation set, and 2 in the test set; 0.1263% of all files; 632.52 s), which users can exclude via the ‘is_synthetic’ field. Two fixes in the test set use a female provider's voice while targeting male public speaker labels and remain explicitly marked. Gender representation across the supplied partitions is a substantial limitation. All eight female human speakers are assigned to the training partition, whereas the validation and test partitions each contain only three male human speakers. Consequently, validation-based model selection and evaluation on held-out human speakers are restricted to male speech. External evaluations using the supplied manifests inherit this gender imbalance in evaluation coverage, and their results cannot be assumed to represent performance across genders. The absence of female human speakers from the validation and test partitions also prevents estimation of differences in recognition accuracy between male and female speakers. The two female-voice synthetic test recordings do not provide evidence of generalization to unseen female human speakers. This partition imbalance therefore limits the interpretation of the reported WER and CER without establishing the direction or magnitude of any performance difference between genders.

The historical partitions are retained to preserve the reproducibility of the frozen nine-model benchmark. An evaluation with female and male speakers represented in every subset would require a separately documented speaker-disjoint partition and renewed model training, fine-tuning, validation, and testing under that protocol. Checkpoints previously trained on speakers reassigned to the test set must not be used to claim generalization to those speakers. Results from a revised partition should be reported separately from the historical benchmark.

Public speaker IDs are pseudonyms; however, the voice recordings may still be potentially identifiable. Finally, the release target, consisting of 104,500 files, differs from the frozen benchmark, consisting of 102,544 files used to generate the saved ASR predictions. Therefore, the technical validation conclusions apply only to the reviewed and frozen benchmark.

## Ethics Statement

Each participant provided informed consent for their speech recordings to be made publicly available. Participants were informed that their participation was entirely voluntary and that they could pause or terminate the recording session at any time. Following completion of the data collection process, all information obtained from the participants was anonymised. The confidentiality and anonymity of the participants’ information were maintained. Each participant approved their post-recording samples for inclusion in the dataset. The study received ethical approval from the relevant research unit at Universitas Sriwijaya prior to data collection. The ethical approval is documented in an official letter issued by the Head of the Research and Community Service Institute of Universitas Sriwijaya.

## CRediT Author Statement

**Ratna Atika:** Data curation, Software, Original draft preparation, Investigation, Visualization; **Suci Dwijayanti:** Writing-Reviewing and Editing, Methodology; **Bhakti Yudho Suprapto:** Supervision, Conceptualization.
